# The role of visual association cortices during response selection processes in interference-modulated response stopping

**DOI:** 10.1093/texcom/tgac050

**Published:** 2023-01-10

**Authors:** Elena Eggert, Filippo Ghin, Ann-Kathrin Stock, Moritz Mückschel, Christian Beste

**Affiliations:** Cognitive Neurophysiology, Department of Child and Adolescent Psychiatry, Faculty of Medicine, TU Dresden, Fetscherstrasse 74, 01309 Dresden, Germany; Faculty of Medicine, University Neuropsychology Center, TU Dresden, Fetscherstrasse 74, 01309 Dresden, Germany; Cognitive Neurophysiology, Department of Child and Adolescent Psychiatry, Faculty of Medicine, TU Dresden, Fetscherstrasse 74, 01309 Dresden, Germany; Faculty of Medicine, University Neuropsychology Center, TU Dresden, Fetscherstrasse 74, 01309 Dresden, Germany; Cognitive Neurophysiology, Department of Child and Adolescent Psychiatry, Faculty of Medicine, TU Dresden, Fetscherstrasse 74, 01309 Dresden, Germany; Faculty of Medicine, University Neuropsychology Center, TU Dresden, Fetscherstrasse 74, 01309 Dresden, Germany; Cognitive Neurophysiology, Department of Child and Adolescent Psychiatry, Faculty of Medicine, TU Dresden, Fetscherstrasse 74, 01309 Dresden, Germany; Faculty of Medicine, University Neuropsychology Center, TU Dresden, Fetscherstrasse 74, 01309 Dresden, Germany; Cognitive Neurophysiology, Department of Child and Adolescent Psychiatry, Faculty of Medicine, TU Dresden, Fetscherstrasse 74, 01309 Dresden, Germany; Faculty of Medicine, University Neuropsychology Center, TU Dresden, Fetscherstrasse 74, 01309 Dresden, Germany

**Keywords:** conflict, EEG, event file, response inhibition, stopping

## Abstract

Response inhibition and the ability to navigate distracting information are both integral parts of cognitive control and are imperative to adaptive behavior in everyday life. Thus far, research has only inconclusively been able to draw inferences regarding the association between response stopping and the effects of interfering information. Using a novel combination of the Simon task and a stop signal task, the current study set out to investigate the behavioral as well as the neurophysiological underpinnings of the relationship between response stopping and interference processing. We tested *n* = 27 healthy individuals and combined temporal EEG signal decomposition with source localization methods to delineate the precise neurophysiological dynamics and functional neuroanatomical structures associated with conflict effects on response stopping. The results showed that stopping performance was compromised by conflicts. Importantly, these behavioral effects were reflected by specific aspects of information coded in the neurophysiological signal, indicating that conflict effects during response stopping are not mediated via purely perceptual processes. Rather, it is the processing of specific, stop-relevant stimulus features in the sensory regions during response selection, which underlies the emergence of conflict effects in response stopping. The findings connect research regarding response stopping with overarching theoretical frameworks of perception–action integration.

## Introduction

Much of contemporary cognitive neuroscience research is dedicated to studying the neural underpinnings of inhibitory control processes, which reflect a significant facet of the executive functions (Diamond 2013). Regarding inhibitory control processes, many different facets can be dissociated (Bari and Robbins 2013), playing a role in many neuropsychiatric disorders (Chmielewski and Beste 2015; Sebastian et al. 2018). These include reactive and proactive inhibitory control processes (Aron 2011; Braver 2012; Meyer and Bucci 2016), with the former commonly studied using stop signal tasks (Logan et al. 1984; Meyer and Bucci 2016; Verbruggen et al. 2019). In stop signal tasks, reactive inhibition can be examined because the instruction to inhibit/stop a response is given after this response has already been initiated. From a behavioral point of view, the critical process consists in how well a participant can interrupt the initiated response, which is measured by estimating the stop signal reaction time (SSRT) (Meyer and Bucci 2016; Verbruggen et al. 2019). A shorter SSRT is associated with better inhibitory control. Recent years have seen a multitude of studies examining various factors which modulate how well individuals can stop their responses (Ridderinkhof et al. 1999; Verbruggen et al. 2004, 2014; Chambers et al. 2007). However, one of the questions which has not been conclusively answered is how distraction and irrelevant information affects stopping performance. Considering the increasing necessity to shift research in the field of response stopping toward real-world generalizability (Hannah and Aron 2021), it is crucial to better understand the effects of interference on response stopping under theoretically clearly interpretable conditions with regard to the neurophysiological foundations.

Previous work has found that distracting information prolongs SSRTs (Ridderinkhof et al. 1999; Verbruggen et al. 2004, 2014; Chambers et al. 2007), which was considered as evidence to support an “attentional” account of reactive control that stresses the importance of sensory detection processes (Verbruggen et al. 2014). To examine these effects, studies have often used flanker stimuli (Verbruggen et al. 2004; Chambers et al. 2007), Stroop stimuli (Ridderinkhof et al. 1999), or perceptual load manipulations (Verbruggen et al. 2014). However, the use of Flanker and Stroop paradigms when studying the effect of interference or distraction on cognitive processes has been criticized (Hommel 2011) for several reasons: In the Stroop task, it is unclear whether the interference effect arises due to a conflict between 2 stimulus-related feature codes or a conflict between the 2 responses mapped onto the semantic meaning of the color word or their print color (Hommel 2011). In the (Eriksen) Flanker task, distractor stimuli and a target stimulus are shown and the participants are required to select a response based on the target stimulus. Interference in this task can thus be due to 1 of 2 relationships: (i) between the coding of the target and the coding of the distractor and (ii) between the responses that are mapped onto the target and distractors (Hommel 2011). Both a Stroop-like and a Flanker-like setting to examine the effects of interference on cognitive processes are difficult to interpret. These problems are not evident when assessing the effect of interference in a Simon task setup, which appears to be more suitable to investigate the interactions between perception and action (Hommel 2011). The effects of the Simon Go/Nogo task are well established (e.g. Sebanz et al. 2003; Chmielewski and Beste 2017; Chmielewski et al. 2018; Dolk and Liepelt 2018). While both Go/Nogo and stop signal paradigms are used to investigate inhibitory control, Go/Nogo tasks measure how well a response can be withheld. Stop signal tasks, on the other hand, specifically examine how well a response which has already been initiated can be interrupted. Thus, both types of task paradigms look at the different aspects of inhibitory control. As Raud et al. (2020) have pointed out, a Nogo stimulus requires action restraint, while a stop signal necessitates action cancelation; the 2 different processes have been found to rely on distinct neural dynamics. Taken together, this emphasizes the novelty and relevance of using a Simon task setup in order to examine response stopping (in a stop signal task), which would uniquely allow for inferences regarding conflict during stimulus–response (S–R) mapping processes in response-stopping performance. Therefore, we developed a novel combination of the Simon task and a stop signal task (“Simon Stop task”) to elucidate the neurophysiological processes and functional neuroanatomical structures associated with the effect of distraction and irrelevant information on stopping performance.

Regarding the neurophysiological processes associated with the conflict effects on response stopping, it is essential to consider the cognitive-theoretical implications of the dynamics in a Simon task (Simon and Rudell 1967; Simon and Small Jr 1969). One of the frameworks that have been put forward to explain the processes in the Simon task is the “Theory of Event Coding (TEC)” (Hommel 1998; Hommel 2011). The central element in TEC is the so-called “event file,” which can be considered to be the mapping of stimuli onto the appropriate response. Thus, an event file contains specifications of the stimuli and the response at the constituent feature level (Hommel 2004, 2009). The constituent features code every stimulus (e.g. shape, spatial location, size, and color). The same is the case for motor responses, which is why stimuli and actions are represented in a common coding format. In the Simon task, a stimulus requires either a left hand or right hand response and is presented on either the left or right side, leading to an event file containing information regarding location and the required motor response. Importantly, the response is to be given irrespective of stimulus location. Consequently, interference effects occur when the side of stimulus presentation is opposed to the side of response (i.e. incongruent Simon trials). In such cases, the event file needs to be reconfigured to allow an appropriate response execution. In a previous study which used Simon task elements in a stop signal task (Verbruggen et al. 2005), results showed that stopping performance was impaired in incongruent trials when they were preceded by a congruent trial. Furthermore, the findings indicated that similar mechanisms underlie the effects of stimulus–stimulus (S–S) compatibility and S–R compatibility on stopping processes. In the present study, it will be of particular interest to determine how interference in a Simon task setup affects inhibitory performance. Therefore, on a statistical level, it will be especially important to examine the interaction of “congruency” (i.e. congruent vs. incongruent), which reflects the Simon/conflict element of the task, and “response” (i.e. Go vs. Stop), which reflects the inhibitory aspect of the task. A significant interaction effect could indicate that the interference processing differs depending on whether response stopping was required and could provide insight regarding the impact of conflict on response stopping.

With regard to the individual event-related potentials (ERP) in the neurophysiological data, research, in general, has shown that the ERP P1 has been associated with early stimulus categorization and the facilitation of sensory processing (Luck et al. 1990; Klimesch 2011) and the N1 has been related to the orientation of attention to task-relevant stimuli (Luck et al. 1990; Herrmann and Knight 2001). In Nogo conditions of Go/Nogo tasks, the N2 has been demonstrated to play a role in the monitoring/processing of conflicts between representations of Go and Nogo and the P3 has been associated with the inhibition of motor processes (Falkenstein et al. 1999; Smith et al. 2008; Huster et al. 2013). Several recent studies have delineated the neurophysiological dynamics of event file coding processes (Petruo et al. 2016; Kleimaker et al. 2020; Opitz et al. 2020; Takacs, Mückschel, et al. 2020; Takacs, Zink, et al. 2020; Takacs, Bluschke, et al. 2021; Takacs, Stock, et al. 2021) and have shown that specific processes reflecting the mapping of stimuli onto the appropriate responses (i.e. event file coding processes) can be isolated in EEG signals.

It has consistently been shown that event file dynamics can be delineated most reliably after performing a temporal signal decomposition using residue iteration decomposition (RIDE) (Ouyang et al. 2015, 2017). RIDE separates the EEG signal into 3 activity clusters with dissociable functional relevance: The R-cluster provides information regarding the response process, the S-cluster refers to information regarding perceptual processing of the stimulus, and the C-cluster is assumed to measure S–R translational aspects (Verleger et al. 2014; Ouyang et al. 2015). Particularly, the C-cluster has been shown to reflect event file coding processes (Opitz et al. 2020; Takacs, Mückschel, et al. 2020; Takacs, Zink, et al. 2020; Prochnow et al. 2021). Moreover, it has been shown that neurophysiological processes reflected during the inhibition of performance, as shown using Go/Nogo tasks, are reflected by the C-cluster (Ouyang et al. 2013). Therefore, we hypothesize that the effects of distraction and interfering information on response stopping are specifically reflected by C-cluster activity.

Furthermore, it appears relevant to determine which functional neuroanatomical structures are associated with the modulation of neurophysiological processes. Considering the literature regarding interference control (Rushworth et al. 2004; Nachev et al. 2008; Mars et al. 2009; Stock et al. 2013; Herz et al. 2014; Mückschel et al. 2016) and stop signal tasks (Aron et al. 2004; Aron 2011; Bari and Robbins 2013), cortical regions, including the right inferior frontal gyrus, the middle frontal gyrus (MFG) as well as superior frontal cortices, may be associated with a difference of C-cluster activity modulation between the interfering and noninterfering conditions. Assuming that there are modulations in these areas, this would suggest that processes which reflect the stopping of a motor response are modulated by the interfering information. Although these aforementioned areas seem very likely to reflect modulations of C-cluster activity, taking the characteristics of event file coding into consideration leads to entirely different hypotheses regarding brain regions associated with C-cluster modulations. Event file dynamics are based on the “identity” of features constituting a stimulus that is bound to a specific response (Hommel 2004). For example, when the manipulation of facial stimuli modulates event file coding, the fusiform face area reflects event file dynamics; when the event file coding is modulated by motion stimuli, completely different areas encode event file dynamics (Kühn et al. 2011). This suggests that, depending on the identity of stimulus features associated with a specific action, the functional neuroanatomy underlying the event file dynamics can change. This is also of interest in the context of the effects of distracting information during stopping, seeing as it has been suggested that sensory detection processes play a central role during stopping (Verbruggen et al. 2014). Therefore, in the current study, we deliberately choose to induce signal stopping processes by a change of the color of letter stimuli presented in a Simon task spatial arrangement. Color processing is a function of the ventral visual processing stream (Goodale and Milner 1992; Chao and Martin 1999; Goodale et al. 2005). In the context of this functional-neuroanatomy-inspired stop process “tagging,” it will be possible to delineate whether the interference effects on stopping processes are due to sensory detection processes or due to processes affecting the motor braking aspects during stopping. If sensory processing is particularly relevant, the differences in stopping processes between congruent and incongruent Simon trials should be reflected in the differences in the activity in the ventral pathway. If motor braking processes play a role, the effects between congruent and incongruent Simon trials during stopping would be more associated with the regions of the frontal cortex.

## Materials and methods

### Participants

In light of the fact that the employed task had never been administered before, the effect sizes needed for an a priori power analysis were not available from previous literature. However, based on the conservative effect size of *f* = 0.3 for both the analysis of the behavioral data and the analysis of the neurophysiological data, an a priori estimation yielded a required sample size of *n* = 26 with an alpha error probability of 5% and a power of 95%. The final sample in the current study consisted of *n* = 27 participants (after exclusions, as described below) in the age range of 18–35 years (16 females; mean age = 25.11, SD = 5.22). All participants reported no neurological or psychiatric illness and were right-handed. Only participants with a stopping probability in the recommended range of 0.25–0.75 (Verbruggen et al. 2019) were included in the sample, leading to the exclusion of 3 participants whose stopping probability was not within this range. In order to establish whether the SSRT effect showed the expected direction (shorter SSRTs in the congruent condition than in the incongruent condition, as evidenced by the behavioral results), the difference between the SSRT in the congruent condition and the SSRT in the incongruent condition was calculated (incongruent subtracted from congruent). One participant with a difference value that was larger than the selected cut-off value of 50 was excluded. Finally, 1 left-handed participant was excluded in order to maintain a homogenous sample with regard to handedness. Handedness was determined based on self-report. The study was approved by the Ethical Committee of the Medical Faculty of the TU Dresden and all participants provided a signed informed consent form prior to taking part in the study. The participants received either a financial reimbursement or course credit for their participation.

### Task

**Fig. 1:**
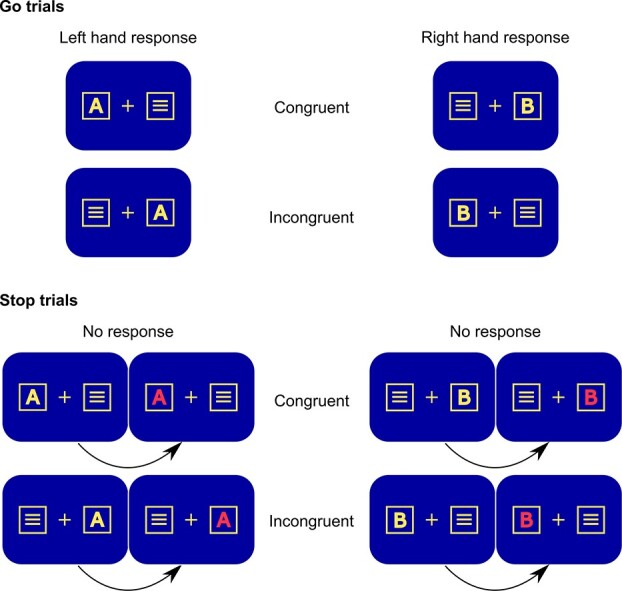
Schematic illustration of the implemented task with all possible stimulus configurations. Congruent and incongruent trials in the Go condition are shown above, while the congruent and incongruent trials in the Stop condition are shown below.

The newly developed Simon Stop task combined a Simon task (Simon and Rudell 1967; Simon and Small Jr 1969) with a stop signal task (Logan et al. 1984). The task was administered to the participants on a computer screen with a blue background color at an approximate distance of ~60 cm. A schematic illustration of the task is shown in Fig. 1.

Throughout the entire duration of the task, a fixation cross was shown in the middle of the computer screen. Two white frame boxes were shown on the left and on the right of the fixation point and on the same horizontal level as the fixation cross at a distance of 0.7 cm. During a trial, a single letter stimulus in the form of either an A or a B in normal font and yellow color appeared in 1 of the 2 boxes, while a distractor stimulus in the form of 3 white horizontal lines was simultaneously displayed in the respective other box. The participants were asked to respond as quickly as possible to the target stimulus. They were instructed to press the left control key with the left index finger upon the presentation of an A and to press the right control key with the right index finger upon the presentation of a B. The response was to be given irrespective of the spatial location of the letter stimulus. Consequently, the presentation of the letter in the box on the same side as the responding hand constituted a congruent trial, while the presentation of the letter in the opposite box in respect to the responding hand constituted an incongruent trial. Thus, there were 2 different conditions that thereby established the Simon element of the task.

In order to incorporate the stop signal component, the task comprised of Go trials and Stop trials. In Go trials, the letter stimulus was displayed unaltered for 1,700 ms or until the participant gave a response. Here, a response was classified as either “correct” or “incorrect,” while a failure to respond was classified as a “miss.” Importantly, in Stop trials, the letter stimulus turned red after a variable delay, thereby constituting a stop signal. In these trials, participants were asked to interrupt (stop) their ongoing response. The stop signal was presented for 1,700 ms. In order to achieve a stopping probability of approximately 0.50 (Verbruggen et al. 2019), the stop signal delay (SSD), i.e. the time period after which the letter stimulus turned red, was adjusted in every trial depending on the response given in the respective preceding trial. When a participant correctly withheld their response in a Stop trial (“correct rejection”), the SSD in the following trial was increased by 50 ms. When a participant failed to stop their initiated response in a Stop trial (“failure to stop”), the SSD in the following trial decreased by 50 ms. The initial SSD duration was set to 250 ms, with a minimum of 50 ms and a maximum of 1,000 ms throughout the duration of the task. A speedup announcement “Bitte versuchen Sie, schneller zu drücken” (German for “Please try to respond faster”) appeared on the screen for 2,000 ms if the mean reaction time of the last 50 trials was below the set minimum reaction time of 450 ms. The intertrial interval had a duration of 1,300 ms.

The entire task had a duration of approximately 40 min and consisted of 9 blocks with a total of 936 trials, 720 of which were Go trials (77%) and 216 of which were Stop trials (23%). Within the Go and the Stop trials, half of the trials were congruent trials and the other half were incongruent trials, respectively (360 congruent and 360 incongruent trials in the Go trials; 108 congruent and 108 incongruent trials in the Stop trials). Each block consisted of 80 Go trials and 24 Stop trials. The order of the trials was randomized. The participants had the option to take breaks between the blocks. Prior to the beginning of the experiment, the participants carried out a standardized exercise of 36 trials with a mix of Go and Stop trials in order to get familiar with the task. During this exercise, the participants received direct feedback following each trial regarding the accuracy of their response.

### E‌EG recording and preprocessing

Using a QuickAmp amplifier and BrainVision Recorder software (Brain Products Inc., Germany), the EEG data were recorded at a sampling rate of 500 Hz from 60 Ag-AgCl electrodes arranged in an equidistant setup. The impedances of the electrodes were maintained <10 Ω. The ground and reference electrodes were positioned at the coordinates θ = 58, ϕ = 78 and θ = 90, ϕ = 90, respectively. The off-line preprocessing of the EEG data was carried out with the Brain Vision Analyzer 2 software package (Brain Products Inc., Germany). In the first step, the sampling rate was changed to 256 Hz and a bandpass filter was applied (0.5–40 Hz using an order of 8 and an additional notch filter at 50 Hz). Electrode channels displaying no activity were discarded from the EEG, and subsequently, the data was rereferenced to an average reference. Any technical artifacts were thereafter removed during a manual inspection of the data. During an independent component analysis (Infomax algorithm), artifacts with recurring effects (e.g. pulse artifacts, blinking, and eye movements) were identified and removed. In a final raw data inspection, remaining artifacts were discarded. Subsequently, the previously removed channels were interpolated topographically.

Individual segments were formed in both the congruent and incongruent conditions for the correct responses in the Go trials and the correct rejections in the Stop trials. The segments were locked to the target stimulus (−2,000 to 2,000 ms) in both the Go trials and the Stop trials. An automated artifact rejection in the time window of 200 ms before and after the target stimulus rejected all segments with amplitudes >100 μV or <−100 μV, or activity over a time period of at least of 100 ms which was <0.5 μV. A baseline correction based on the mean activity in the time period of −200 ms to 0 ms before the stimulus onset was performed.

### Residue iteration decomposition

The EEG data were decomposed by using RIDE (Ouyang et al. 2013; Ouyang et al. 2015). The application of RIDE enables the derivation of separate clusters within ERPs regarding stimulus-related properties (“S-cluster”), response-related information (“R-cluster”), and S–R translational aspects (“C-cluster”) (Verleger et al. 2014; Ouyang et al. 2017). To this end, RIDE decomposes single-trial ERPs in an iterative manner, resulting in the aforementioned different clusters of components based on their timing and variability, which thus are related to different phases of information processing (Ouyang et al. 2013, 2015). Seeing as every electrode channel is decomposed individually, RIDE provides a high sensitivity to the channel-specific latency variability information (Ouyang et al. 2013, 2015). Latency information regarding the stimulus onset is used to derive the S-cluster and the R-cluster, while the latency information of the C-cluster is repeatedly determined and improved in every trial. In order to obtain an estimation of the S-cluster, the RIDE algorithm subtracts C and R from every single trial and subsequently establishes an alignment of the residual of all trials to the S-cluster latency information, resulting in the median waveform for every time point in the interval of the S-cluster. The C-cluster and the R-cluster are derived in the same manner. In the current study, only the C-cluster and the S-cluster were examined, seeing as the reliable analysis of the R-cluster is based on motor response execution, which is expected not to occur in trials where motor inhibitory control is required (Ouyang et al. 2013). Using RIDE, and when not modeling the R-cluster, activity that would otherwise have been allotted to the R-cluster is also represented in the C-cluster (Ouyang et al. 2013). RIDE was carried out with the RIDE toolbox (manual available at http://cns.hkbu.edu.hk/RIDE.htm) in MATLAB (MathWorks, Inc., Natick, MA). Time windows were defined to extract the waveforms related to the different clusters. For the S-cluster, which was expected to cover the classic ERPs from P1 to N2, a time window of 200 ms prior and 600 ms after the presentation of the stimulus was applied. For the C-cluster, which was expected to capture the P3-component, a time window of 200–800 ms poststimulus presentation was determined. These time markers were used to iteratively decompose ERP components according to L1 norm, thereby establishing median waveforms. Upon obtaining the RIDE clusters, the components corresponding to classic ERPs were quantified. The electrodes P7 and P8 were chosen to analyze the P1 and N1 components in the S-cluster in the time windows of 100–120 ms and 160–180 ms, respectively. The FCz was specified to examine the N2 component in the S-cluster in the time window of 290–320 ms. The electrodes FCz and Pz were chosen to quantify the P3 component in the C-cluster in the time window of 530–650 ms. The appropriate time windows and electrodes were selected based on the visual inspection of the grand averages of each cluster. For the P3, the time window was further based on the difference plot between the congruent and incongruent correct rejection Stop trials.

### Source localization

The standard low-resolution brain electromagnetic tomography algorithm (sLORETA) (Pascual-Marqui 2002) was applied to the RIDE-decomposed data in order to investigate the sources of congruency effects in Stop signal trials and the main effect Go versus Stop. Since only the C-cluster revealed such effects in the electrode level analysis, only the C-cluster was examined. As an EEG source localization method, sLORETA comes with limitations such as low spatial resolution and the potential for spatial blurring (Jatoi et al. 2014). However, sLORETA allows for a more precise localization of deep brain structures than other localization methods (Grech et al. 2008), reveals valuable information about brain processes in combination with structural imaging data (Ocklenburg et al. 2018), converges with brain stimulation effects (Dippel and Beste 2015), and offers a solution to the inverse problem, thereby avoiding localization bias (Pascual-Marqui 2002; Marco-Pallarés et al. 2005; Sekihara et al. 2005). After dividing the intracerebral volume into 6,239 voxels at a spatial resolution of 5 mm within a 3-shell spherical head model, the calculation of the standardized current density for each voxel is performed based on a MNI152 head model template (Mazziotta et al. 2001). Past research involving combined functional magnetic resonance imaging/EEG and transcranial magnetic stimulation/EEG have provided evidence for the validity of sLORETA results (Sekihara et al. 2005). Based on statistical nonparametric mapping, voxel-wise randomization tests with 2,500 permutations were carried out in order to compute the statistics of the sLORETA sources. Significant differences (*P* < 0.05) between the voxel locations of the contrasted conditions were displayed in the MNI-brain.

## Results

### Behavioral data

Repeated-measures ANOVAs with the factors “position” (left vs. right) and “congruency” (congruent vs. incongruent) were performed in order to analyze the obtained behavioral measures in Go trials and Stop trials. When needed, a Greenhouse–Geisser correction was applied in order to take into consideration a potential lack of sphericity. When variables were not normally distributed (as examined using Kolmogorov–Smirnov tests), Wilcoxon tests were used for post hoc analyses.

#### Go trials

For the accuracy rate in the Go trials, the data revealed a main effect of “congruency,” with participants showing a higher accuracy rate in congruent trials (97.17% ± 4.06) compared to incongruent trials (93.67% ± 6.89; *F*(1, 26) = 15.53, *P* < 0.001, ƞ*_p_*^2^ = 0.37). Overall, there was an accuracy rate of 95.52% (± 5.15). Furthermore, the analysis demonstrated an interaction effect for “position x congruency” (*F*(1, 26) = 7.91, *P* = 0.009, ƞ*_p_*^2^ = 0.23). However, post hoc Wilcoxon tests revealed that there was no difference in the accuracy rate between the positions of the stimulus presentation for the congruent trials (*Z* = −1.097, *P* = 0.273) or the incongruent trials (*Z* = −1.097, *P* = 0.235).

For the reaction times in the Go trials, a main effect of “congruency” was shown: Faster reaction times were evident in congruent trials (507 ms ± 169) compared to incongruent trials (532 ms ± 162; *F*(1, 26) = 38.81, *P* < 0.001, ƞ*_p_*^2^ = 0.60). Furthermore, the analysis revealed a marginally significant main effect of “position” (*F*(1, 26) = 3.37, *P* = 0.078, ƞ*_p_*^2^ = 0.12), with higher reaction times for the left side of stimulus presentation (524 ms ± 167) than for the right side of stimulus presentation (515 ms ± 164). There were no other significant effects for the accuracy or the reaction times in the Go trials (all *F*s < 0.58, all *P*s > 0.452).

#### Stop trials

**Fig. 2:**
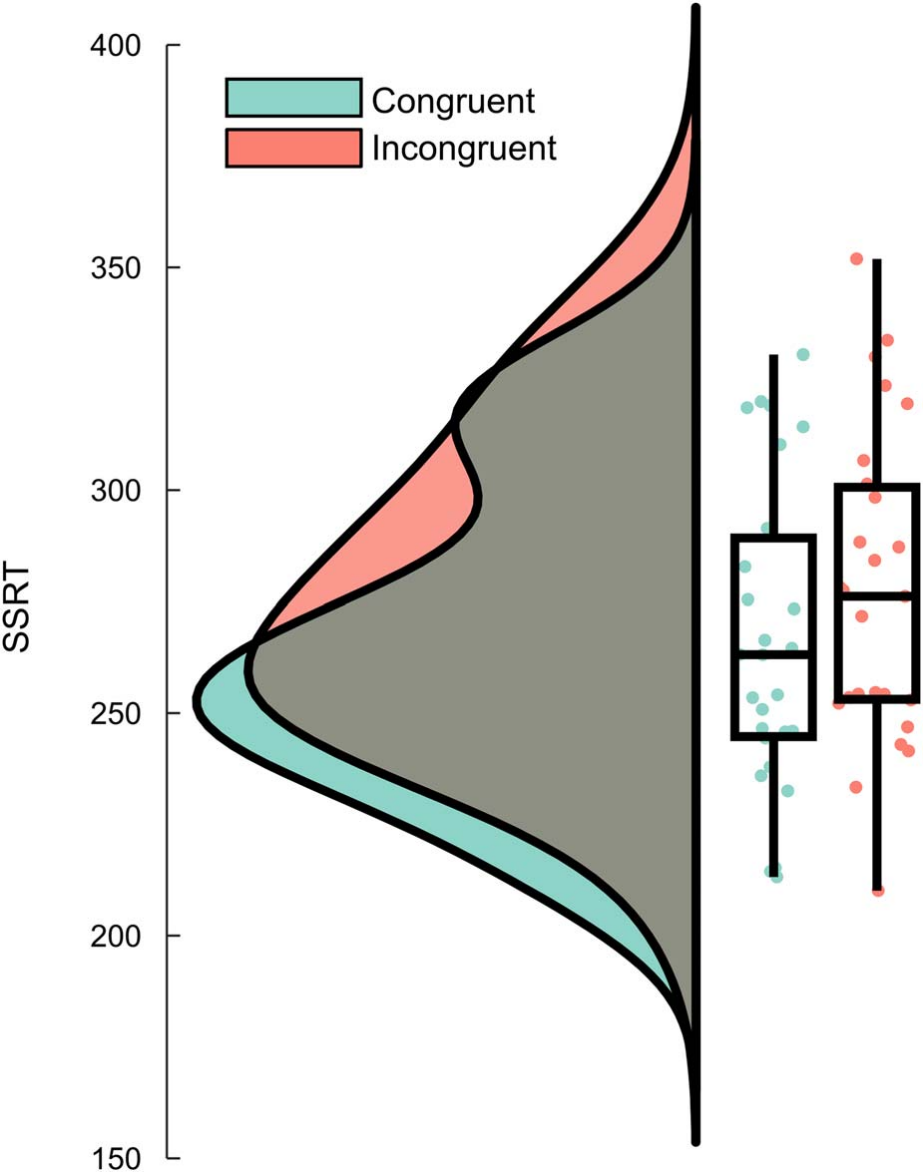
SSRT data for congruent and incongruent Stop trials. For each condition, a boxplot, and the individual data points as well as the probability distribution are given.

Overall, there was a mean error rate (i.e. probability of responding in a Stop trial) of 50.69% (±6.32) and the mean SSD was 248 ms. The SSRT was calculated based on the mean estimation method. The analysis revealed a main effect of “congruency,” with participants displaying a shorter SSRT in congruent trials (266 ms ± 35) than in incongruent trials (277 ms ± 35; *F*(1, 26) = 9.00, *P* = 0.006, ƞ*_p_*^2^ = 0.26). The stop signal reaction-time data for congruent and incongruent trials are shown in Fig. 2. There were no other significant effects for the SSRT (all *F*s < 0.25, all *P*s > 0.62). As traditionally shown in stop signal tasks (Schall et al. 2017), participants demonstrated shorter reaction times in their failures to stop during the Stop trials (460 ms ± 133) than in the correct responses during the Go trials (533 ms ± 167; *Z* = −4.54, *P* < 0.001).

In the Stop trials, there was a main effect of “congruency” for the accuracy rate, with participants showing a higher error rate (i.e. failures to stop their response) in the congruent trials (51.17% ± 6.75) than in the incongruent trials (50.21% ± 6.00; *F*(1, 26) = 6.57, *P* = 0.017, ƞ*_p_*^2^ = 0.20). Moreover, the reaction time data of the failures to stop showed a main effect of “congruency,” with faster responses in the congruent trials (447 ms ± 135) than in the incongruent trials (474 ms ± 132; *F*(1, 26) = 69.68, *P* < 0.001, ƞ*_p_*^2^ = 0.73). There was also a main effect of “position”: The reaction times of the failures to stop were longer in trials when the stimulus was presented on the left side (467 ms ± 138) than when it was presented on the right side (454 ms ± 129; *F*(1, 26) = 10.30, *P* = 0.004, ƞ*_p_*^2^ = 0.28). There were no other significant effects for the accuracy and the reaction times of the failures to stop in the Stop trials (all *F*s < 0.84, all *P*s > 0.368).

### Neurophysiological data

Repeated-measures ANOVAs with the factors “response” (Go vs. Stop) and “congruency” (congruent vs. incongruent) were calculated in order to analyze the RIDE-ERPs in the RIDE-decomposed data. A Greenhouse–Geisser correction was applied when necessary to account for lack of sphericity. In the current context, the interaction “response × congruency” was of particular interest. Therefore, the respective results were validated by means of Bayesian statistics using the template established by Masson (2011) according to the recommendations by Wagenmakers (2007). Based on the observed data, the analysis establishes the probability of the null hypothesis being true (*P*(H0|D)). Probability values <0.5 suggest a stronger likelihood for the alternative hypothesis (i.e. presence of interaction) to be true compared to the null hypothesis (i.e. lack of interaction). Values in the range of 0.5–0.75 reflect weak evidence, values in the range of 0.75–0.95 indicate positive evidence, and values in the range of 0.95–0.99 suggest strong evidence for the null hypothesis to be true (Raftery 1995). While Bayesian statistics could provide information on whether the findings of the frequentist approach could be corroborated or not, our final interpretation of the results was based on the ANOVA findings.

#### S-cluster

The analysis of the P1 amplitude in the time interval of 100–120 ms revealed no significant effect at the P7 (all *F*s < 0.76, all *P*s > 0.392) or at the P8 (all *F*s < 2.64, all *P*s > 0.116). Bayesian analysis regarding the interaction of “congruency x response” at the P7- and the P8-obtained values of *P*(H0|D) = 0.867 and *P*(H0|D) = 0.825, respectively, indicating that the null hypothesis is likely to be true, which confirms the ANOVA results.

The analysis of the N1 amplitude in the time interval of 160–180 ms showed a marginally significant main effect of “congruency” at the P7 (*F*(1, 26) = 3.98, *P* = 0.057, ƞ*_p_*^2^ = 0.13), with a larger (i.e. more negative) amplitude for the incongruent trials (−3.53 ± 2.80 μV/m^2^) than for the congruent trials (−3.27 ± 2.43 μV/m^2^). None of the other effects at the P7 were significant (all *F*s < 2.22, all *P*s > 0.148). Bayesian analysis regarding the interaction of “congruency × response” showed a value of *P*(H0|D) = 0.246, indicating that the alternative hypothesis is likely to be true, which is not in line with the ANOVA results. At the P8, on the other hand, the analysis of the N1 revealed an interaction effect of “congruency × response” (*F*(1, 26) = 5.96, *P* = 0.022, ƞ*_p_*^2^ = 0.19). However, post hoc tests revealed no significant difference between the congruent and incongruent trials in the Stop condition (*t*(26) = 1.76, *P* = 0.090) or the Go condition (*t*(26) = −1.19, *P* = 0.247). None of the main effects at the P8 were significant (all *F*s < 0.82, all *P*s > 0.372). Bayesian analysis regarding the interaction of “congruency × response” at the P8 revealed a value of *P*(H0|D) = 0.002, indicating that the alternative hypothesis is likely to be true, corroborating the ANOVA results.

**Fig. 3:**
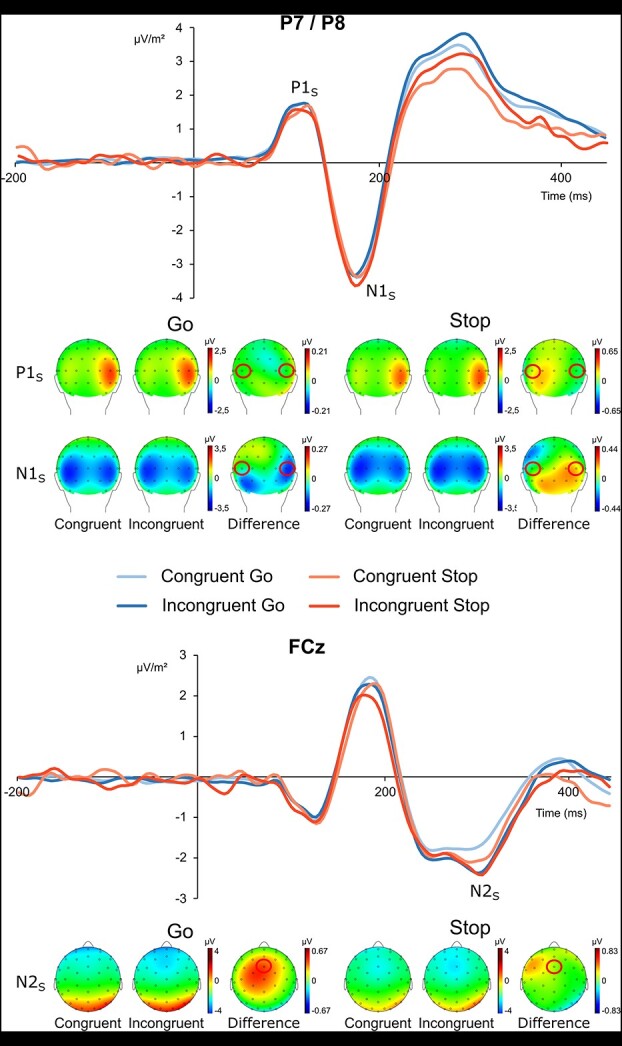
Illustration of the P1 (100–120 ms), N1 (160–180 ms), and N2 (290–320 ms) components of the RIDE-decomposed data in the S-cluster along with the corresponding topographic plots of the activation pattern as well as the difference waves between the congruent and incongruent Go and Stop trials, respectively. The P1 and N1 were averaged across the electrodes P7 and P8, while the N2 was shown at electrode FCz. Time point zero reflects the time point of stimulus presentation.

For the N2 amplitude at the FCz in the time interval of 290–320 ms, a repeated-measures ANOVA showed a main effect of “congruency” (*F*(1, 26) = 14.70, *P* < 0.001, ƞ*_p_*^2^ = 0.36), with a higher amplitude (i.e. less negative) in congruent trials (−1.88 ± 2.12 μV/m^2^) than in incongruent trials (−2.29 ± 2.20 μV/m^2^). No other effect was significant (all *F*s < 2.51, all *P*s > 0.125). Bayesian analysis regarding the interaction of “congruency × response” at the FCz revealed a value of *P*(H0|D) = 0.177, indicating that the alternative hypothesis is likely to be true, which is not in line with the ANOVA results. The P1, N1, and N2 ERPs are shown in Fig. 3. For an analysis of the N2pc in the S-cluster, please refer to the supplemental material.

#### C-cluster

For the P3 amplitude at the FCz in the time interval of 530–650 ms, a repeated-measures ANOVA revealed a main effect of “congruency” (*F*(1, 26) = 16.18, *P* < 0.001, ƞ*_p_*^2^ = 0.38), with a higher amplitude in the incongruent trials (0.73 ± 2.02 μV/m^2^) than in the congruent trials (0.17 ± 2.00 μV/m^2^). There was no significant main effect for “response” (*F*(1, 26) = 0.48, *P* = 0.495, ƞ*_p_*^2^ = 0.018). Furthermore, the analysis showed an interaction effect of the factors “congruency” and “response” (*F*(1, 26) = 5.67, *P* = 0.025, ƞ*_p_*^2^ = 0.18). Post hoc tests revealed a higher amplitude in the incongruent trials (1.09 ± 3.06 μV/m^2^) than in the congruent trials (0.18 ± 2.93 μV/m^2^) in the Stop condition (*t(*26) = −3.75, *P* < 0.001), while there was no difference between the congruent and incongruent trials in the Go condition (congruent: 0.16 ± 1.79 μV/m^2^, incongruent: 0.37 ± 1.82 μV/m^2^; *t*(26) = −1.39, *P* = 0.176). Bayesian analysis regarding the interaction of “congruency × response” at the FCz revealed a value of *P*(H0|D) = 0.003, suggesting that the alternative hypothesis is likely to be true, corroborating the ANOVA results. Using sLORETA, we contrasted congruent with incongruent Stop trials. The results show that activity modulations were associated with regions in the left inferior temporal gyrus (BA19; MNI (−51, −65, −5)), the left middle occipital gyrus (BA19; MNI (−51, −61, −11)), the left middle temporal gyrus (BA37; MNI (−51, −63, 0)) the right inferior (BA22; MNI (60, −49, −19))/middle temporal gyrus (BA37; MNI (60, −53, −7)), and the right parahippocampal gyrus (BA36; MNI (18, −39, −7)). The activation was stronger in the incongruent Stop trials than in the congruent Stop trials.

**Fig. 4:**
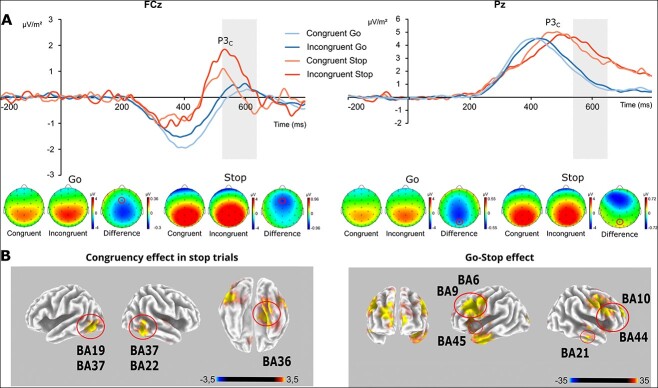
A) Illustration of the P3 component (530–650 ms) of the RIDE-decomposed data in the C-cluster at the FCz and the Pz along with the corresponding topographic plots of the activation pattern as well as the difference waves between congruent and incongruent Go and Stop trials, respectively. Time point zero reflects the time point of stimulus presentation. B) Results of the sLORETA analysis illustrating the contrast between congruent and incongruent condition in the Stop trials (left) and the contrast between the Go trials and the Stop trials (right) along with the respective Brodman areas showing the difference in activity modulation.

For the P3 amplitude at electrode Pz in the time interval of 530–650 ms, a repeated-measures ANOVA revealed a main effect of “congruency” (*F*(1, 26) = 7.40, *P* = 0.011, ƞ*_p_*^2^ = 0.22), with a higher amplitude in incongruent trials (2.81 ± 1.88 μV/m^2^) than in congruent trials (2.42 ± 1.86 μV/m^2^). Furthermore, there was a main effect of “response” (*F*(1, 26) = 16.94, *P* < 0.001, ƞ*_p_*^2^ = 0.40), with a higher amplitude in the Stop trials (3.72 ± 2.85 μV/m^2^) than in the Go trials (1.51 ± 1.58 μV/m^2^). The interaction effect “congruency × response” was not significant (*F*(1, 26) = 0.32, *P* = 0.577, ƞ*_p_*^2^ = 0.01). Bayesian analysis regarding the interaction of “congruency × response” at the P8 revealed a value of *P*(H0|D) = 0.846, indicating that the null hypothesis is likely to be true, corroborating the ANOVA results. According to the sLORETA analysis, the difference between the Go and Stop trials was associated with the activation differences in the right precentral gyrus (BA6; MNI (47, −5, 41), the right middle (BA10; MNI (27, 55, 24)) and right medial frontal regions (BA9; MNI (7, 44, 24)), and right inferior frontal regions (BA44; MNI (54, 14, 24); BA9; MNI (53, 16, 24)) and the right inferior temporal gyrus (BA21; MNI (66, −14, −20). In the left hemisphere, activation differences were found in the precentral gyrus (BA6; MNI (−47, −1, 41)), the MFG (BA9; MNI (−44, 3, 41)), the inferior frontal gyrus (BA45; MNI (−49, 20, 22)), and the medial frontal gyrus (BA9; MNI (−10, 40, 22)). In all regions, there was a higher activation in the Stop trials than in the Go trials. The P3 ERPs at the FCz and the Pz are shown in Fig. 4.

## Discussion

In the current study, we investigated the neurophysiological and functional neuroanatomical underpinnings of interference effects during response stopping. To this end, we designed a novel experimental paradigm combining a Simon task with a Stop signal task. Based on previous research (Hommel 2011), the Simon task reflects a theoretically stringent vehicle to examine interactions between perception and action and the effects of interference on other cognitive functions (e.g. response stopping). The combination of a Simon task and a Stop signal task resulted in 2 relevant trial types in which stopping had to be performed—congruent and incongruent trials. The behavioral data showed that stopping performance (as measured using the SSRT) was compromised in incongruent Simon task trials compared to congruent Simon task trials. Previous studies using different experimental approaches to study the interference effects on stopping performance have also reported, which compromised response stopping when conflicting information was evident (Ridderinkhof et al. 1999; Verbruggen et al. 2004; Chambers et al. 2007). Since the current study used a different experimental approach and still yielded the same results, the findings conceptually replicate interference effects on response stopping performance. In contrast to Verbruggen et al. (2005), our findings showed an impaired stopping performance even when the congruency condition in the preceding trial was disregarded.

Several classes of interference effects have been distinguished, 2 of which are based on S–S and S–R conflicts (Kornblum et al. 1990; Simon and Berbaum 1990; Hommel 1997). An S–S conflict is based on a similarity between stimulus dimensions, while an S–R conflict is associated with a relevant stimulus feature being mapped onto a response (Hasshim and Parris 2015). While Flanker and Stroop paradigms have been classified as S–S conflict tasks which may impede the differentiation between stimulus-related and response-related information, the Simon task is considered as an S–R conflict paradigm (Kornblum et al. 1990). In this context, thus, the current results show that conflicts or interferences during the S–R mapping processes impair the action-stopping performance. By using a Simon setup for the newly designed task in the current study, the effects can be explained by means of stringent theoretical frameworks such as the TEC (Hommel 2009). According to TEC, interference effects in incongruent Simon trials emerge because the event file specifies that the mappings of stimulus features to response features need to be reconfigured to respond appropriately. Since this is a time-consuming process (Hommel 2009), longer reaction times occur in incongruent than congruent Simon trials. The current data show that this event file reconfiguration process interferes with the process of response stopping, as evidenced by longer SSRTs in incongruent than congruent trials. However, it remains unclear which processes during response stopping are affected by the interference and reconfiguration of event files.

The neurophysiological data were analyzed for congruency effects during stopping performance after performing RIDE (Ouyang et al. 2015). This was a theory-driven decision in the data analysis strategy because several recent studies have consistently shown that the event file dynamics can be delineated most reliably after performing RIDE and that event file dynamics are specifically reflected by the C-cluster (Petruo et al. 2016; Kleimaker et al. 2020; Opitz et al. 2020; Takacs, Mückschel, et al. 2020; Takacs, Zink, et al. 2020; Takacs, Bluschke, et al. 2021; Takacs, Stock, et al. 2021). The approach seems feasible because the EEG signal comprises various aspects of information from multiple functional neuroanatomical sources (Nunez et al. 1997; Huster et al. 2015; Stock et al. 2017). Previous research has demonstrated a congruency effect at the P3 in a classic Simon task, with increased activity in the incongruent trials than in the congruent trials (Wessel et al. 2019). The present study shows the same data pattern within the Stop trials: During incongruent Stop trials, the C-cluster was larger (more positive) in a time window between 530 and 650 ms after stimulus presentation than during the congruent Stop trials. During inhibitory control (e.g. during Go/Nogo tasks), the C-cluster reflects processes that are usually observed in the Nogo-P3 time window when examining the standard ERPs (Ouyang et al. 2013; Chmielewski et al. 2018), which have been suggested to reflect processes that are necessary to stop a motor response (Wessel and Aron 2015). Increased demands on inhibitory control processes have often been associated with an enlarged P3 (Huster et al. 2013). Therefore, the larger (more positive) C-cluster amplitude at frontal electrodes in incongruent than in congruent Stop trials suggests a more effortful implementation of stopping processes. Notably, the stop signal was presented (i.e. the letter stimulus turned red) only after a specific time period had passed. Before the stop signal was shown, a stimulus configuration was shown to trigger a response. Therefore, it is likely that participants had already started to reconfigure their event file in preparation to respond when the stop signal was presented. The already ongoing event file reconfiguration process likely made it more demanding to implement the stopping processes.

More recent views of the brain as a predictive organ based on generative models of brain functions have emphasized the role of the interplay of bottom-up and top-down processes in functional integration/specialization (Friston 2002, 2005). Here, it is postulated that these forward/backward connections enable the brain to make inferences regarding the cause of sensory inputs and thereby facilitate the development of prior expectations/predictions which then guide behavior (Friston 2002, 2005). Thus, these recognition dynamics entail the navigation of expected input as well as prediction errors and can provide insights regarding cortical organization (Friston 2005). Generally, these views suggest that it is the minimization of the brain’s free energy which underlies perceptual inference and learning (Friston 2005, 2009; Friston et al. 2017). Applying Friston’s (2005) theory of cortical responses to the present task paradigm, it could be argued that specific task-setting parameters, such as number of factors, perceptual categories, and flexibility of the task context, have an influence on the associated neural substrates as well as the measured cortical responses and thus impact the learning and inference processes. Specifically, for example, it appears plausible that the infrequent occurrence of stop signals in the incongruent task condition may lead to a larger extent of (expected) surprise, which then in turn might play role in the development of predictions regarding upcoming trials. With regard to the P3, it has been established that while P3-like responses related to perceptual learning originate from mainly parietal regions (Hamamé et al. 2011; Ahmadi et al. 2018), the process of updating of beliefs evokes mainly anterior P3-like responses (Kolossa et al. 2015). The current analysis identified P3 responses at both a frontocentral (FCz) and at a midline parietal electrode (Pz), indicating that the measured P3 responses reflect a mixture of both P3 responses that are preserved and disrupted after frontal lobe lesions (Knight 1997).

In the S-cluster, the findings showed an interaction effect at the N1, although subsequent analysis failed to reveal a difference between the congruent and incongruent conditions in both the Stop trials and the Go trials. Furthermore, there was a main effect of congruency, with a larger (more negative) amplitude in incongruent trials than in the congruent trials at the N2, which is in line with previous literature concerning conflict tasks (Folstein and Van Petten 2008). However, the results from the ANOVA and the Bayesian analysis were not congruent for the S-cluster data, which is why we do not interpret these results in conceptual terms. Opposed to the C-cluster, there were no effects between the incongruent and congruent Stop trials in the S-cluster. Since the S-cluster has been suggested to reflect bottom-up (environmentally driven) perceptual processes (Ouyang et al. 2011, 2015), these findings suggest that purely sensory processing cannot account for the interference modulations on stopping processes. Crucially, however, this does not imply that stimulus-related properties are irrelevant to the observed modulations in the C-cluster. As previously mentioned, the event file contains both stimulus feature codes and response feature codes (Hommel 2009), and the C-cluster has been conceptualized to reflect the translational processes between stimulus evaluation and responding (Verleger et al. 2014; Ouyang et al. 2015, 2017). Therefore, both stimulus- and response-related information are evident in the C-cluster and in the neurophysiological signal, which reflects the modulations of stopping processes by interfering information. Problems in the event file processing and implementation of a new event file (e.g. for stopping) due to interference can thus stem from the stimulus feature level in the event file, the motor feature level in the event file or a combination of both levels.

Intriguingly, the current sLORETA results show that areas in the ventral visual processing stream, i.e. the left inferior temporal gyrus (BA19), the left middle temporal and occipital gyrus (BA37), the right inferior (BA37) and middle temporal gyrus (BA22), and the right parahippocampal gyrus (BA36) were associated with congruency-dependent modulations of the C-cluster activity during Stop trials. In the experiment, the stop signal was implemented by changing the color of the letter stimulus from yellow to red. Color processing is a domain of the ventral visual pathway (Goodale and Milner 1992; Chao and Martin 1999; Goodale et al. 2005), and the processing of the critical change in stimulus features—prompting the reconfiguration of a previously established event file in order to stop this response—is therefore dependent on ventral stream functioning. As outlined above, event files need to be reconfigured during incongruent Simon trials because some features of the stimulus (i.e. the spatial position of the letter) are irrelevant to the response. Incongruent Simon trials, therefore, necessitate a reweighting of the relative importance of the “spatial position feature” and the “letter identity feature,” making only the latter relevant to response execution. When these processes are ongoing and a novel stimulus feature emerges in this dynamic (i.e. the change in color signaling to stop a response), the likelihood is high that this novel stimulus feature cannot or can only slowly be accounted for, then reflected by longer SSRTs. The activation in the ventral pathway during event file reconfiguration may reflect the processes of attentional orienting to the stimulus features (Corbetta et al. 2008; Hampshire et al. 2010). However, since the modulatory effects were not evident in the stimulus-related S-cluster, but rather during perception–action integration processes as captured by the C-cluster, it appears unlikely that attentional orienting plays the primary role in the modulation of response stopping.

The current findings suggest that it is mainly the stimulus feature level and processes within an event file which are essential for the emergence of interference effects during response interruption/stopping. Interestingly, no significant activity differences in the inferior frontal, dorsolateral frontal, and superior frontal cortices were evident when contrasting the congruent and incongruent Stop trials. Particularly, the inferior frontal areas have been suggested to be associated with motor braking functions (Aron et al. 2015). The lack of significant activity modulations that were observed may thus indicate that these motor braking processes, in contrast to the stimulus-related processes discussed before, may only play a minor role in the observed effects of interference on stopping. Interestingly, recent theoretical accounts on stopping Diesburg and Wessel (2021) argue that previously established distinctions between the contribution of attentional orienting and motor inhibition processes are almost impossible to disentangle. This is also true from the perspective of the TEC framework, stating that stimulus and response codes are represented in a common coding format (i.e. the event file) (Hommel et al. 2001; Hommel 2009). The current findings corroborate this, since specifically the C-cluster activity, which is very likely to reflect event file dynamics (Petruo et al. 2016; Kleimaker et al. 2020; Opitz et al. 2020; Takacs, Mückschel, et al. 2020; Takacs, Zink, et al. 2020; Prochnow et al. 2021; Takacs, Bluschke, et al. 2021; Takacs, Stock, et al. 2021), was modulated by interference effects during response stopping. The observed effects may also be explained in terms of hybrid frameworks combining bottom-up associative accounts with top-down control-based aspects (Blais et al. 2007; Blais and Verguts 2012; Egner 2017). The congruency effect found in the Stop trials in the C-cluster constitutes bottom-up feature integration based on the change of color of the target stimulus. On the other hand, the Go-Stop effect in the C-cluster reflects top-down inhibitory control, suggesting that both bottom-up and top-down processes played a role in the perception–action integration dynamics in the present study. Furthermore, the findings may be considered in light of the diffusion model for conflict tasks developed by Ulrich et al. (2015) as an extension of the diffusion decision model (Ratcliff 1978), which postulates that information from both a task-relevant route and from an automatic task-irrelevant route are integrated in a single process in conflict tasks (Salzer et al. 2017). In the Simon task, the differentiation of these 2 routes in the dual-route model has been proposed to be reflected in the dorsal and ventral pathways, with the parallel processing of information in these pathways leading to the response conflict in incongruent conditions (Salzer et al. 2017). The ventral pathway is assumed to process the task-relevant features, which is in line with our findings showing the association of the ventral processing stream with the Stop trial congruency effect in the C-cluster.

The findings also support the notion that the distinction between stimulus-related and motor-related processes is nearly impossible (Diesburg and Wessel 2021). The question is not whether stimulus-related “or” motor-related processes are essential for response stopping and modulatory effects. Instead, it is crucial to consider the relative importance of stimulus-related and motor-related processes or the role of stimulus-feature processing during response selection. The modulatory effects at the focus of this study indicate that the stimulus-related aspect is more critical than the motor-related aspect during response selection. When contrasting Go trials against Stop trials using sLORETA, there were not only activity differences in the temporal cortices but also in the middle and medial frontal regions and in the right/left inferior frontal regions, revealing higher activity during stopping trials than Go trials. This pattern of activity reflects the cortical response inhibition network (Bari and Robbins 2013) and is in line with findings that the right inferior frontal cortex is involved in stopping a response (Aron et al. 2015). This finding indirectly validates the sLORETA findings and the discussion of the findings obtained for the contrast between congruent and incongruent Stop trials. With regard to the motor response, the present task paradigm involved proactive as well as reactive control: Proactive motor inhibition is reflected in the anticipation of stopping, while reactive motor inhibition is reflected in the inhibitory response upon the presentation of a stop signal (Wessel 2018). It has been suggested to implement surprise-based paradigms to examine pure reactive inhibitory control and thereby more adequately capture mechanisms underlying real-world action stopping (Wessel 2018). As a limitation of the current study, the amount of (expected) surprise was not estimated. It appears feasible for future studies to examine the association of interference and response stopping in the context of information theory/processing.

## Conclusion

In summary, we investigated the neurophysiological and functional neuroanatomical underpinnings of interference effects during response stopping using a novel experimental paradigm combining a Simon task with a Stop Signal task. This enabled a theoretically stringent, mechanistic interpretation of the effects using the TEC framework. We showed robust conflict effects on response stopping. These behavioral effects were reflected by specific aspects of information coded in the neurophysiological signal, showing that conflict effects during response stopping are not mediated via purely perceptual processes. Rather, the data show that it is the processing of specific, stop-relevant stimulus-features in visual association cortices during response selection which underlies the emergence of conflict effects in response stopping. The results synthesize conceptual distinctions regarding the importance of perceptual/attentional or motor processes in response stopping and connect research on response stopping with more overarching frameworks of perception–action integration.

## Funding

This work was supported by grants from the Deutsche Forschungsgemeinschaft (DFG) (FOR 2698 and FOR 2790).

*Conflict of interest statement*: None declared.

## Code availability

Published software packages were used and they are indicated in the Materials and methods section. Scripts for data analysis will be made available upon reasonable request.

## Data availability

Data will be made available upon reasonable request.

## Supplementary Material

Supplemental_Material_tgac050
