# Supplementary material for: The role of visual association cortices during response selection processes in interference-modulated response stopping
Source: Cereb Cortex Commun. 2023 Jan 10;4(1):tgac050. doi: 10.1093/texcom/tgac050 (PMC9837466; doi:10.1093/texcom/tgac050)
Supplement: Supplemental_Material_tgac050 [file supplemental_material_tgac050.docx]

**Supplemental Material**

**The role of visual association cortices during response selection processes in interference-modulated response stopping**

Elena Eggert, Filippo Ghin, Ann-Kathrin Stock, Moritz Mückschel, Christian Beste

**Neurophysiological analysis of the N2pc (Stop trials)**

In order to examine lateralized ERPs in the Stop trials in the S-cluster, we calculated the Stop trials differences waves between trials when the stimulus was presented on the left and right side (for both the congruent trials and the incongruent trials) based on the electrodes P7 and P8. Based on the visual inspection of the peaks in these difference waves, we selected a time window of 205-225 ms after the onset of the target stimulus (a time period which corresponds to the N2). After exporting the data, we calculated the N2pc for all conditions, substracting the ipsilateral potential from the contralateral potential (P8 – P7 for the left side and P7 – P8 for the right side, for both the congruent and the incongruent conditions). Subsequently, one dataset had to be excluded due to outliers in various variables, leading to an analysis with n = 26 participants.

The within-subjects ANOVA with the factors “congruency” (congruent vs. incongruent) and “position” (left vs. right) showed a significant main effect of “congruency” (*F*(1,25) = 27.83, *p* < .001, $\eta_{p}^{2}$ = .527), with a more negative amplitude in in the congruent trials (-1.26 ± 1.46 µV/m²) than in the incongruent trials (1.32 ± 1.64 µV/m²). The main effect of “position” and the interaction effect “congruency x position” did not reach significance (all *F* < 2.50, all *p* > .127).


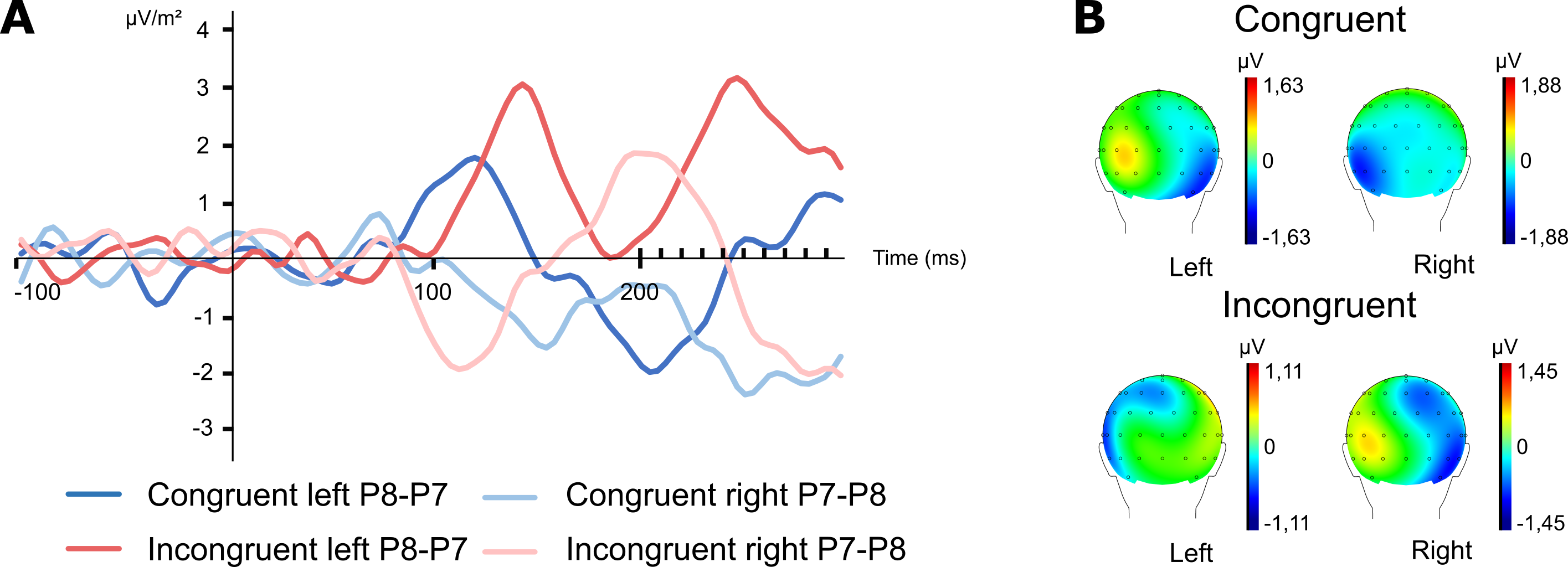


Figure S1. A) Illustration of the N2pc (205-225 ms) component of the RIDE-decomposed data in the S-cluster based on the P7 and P8 electrodes for congruent and incongruent Stop trials with stimulus presentation on the left side and on the right side, respectively. Time point zero reflects the time point of stimulus presentation. B) Topographic plots of the 205-225 ms time window for congruent and incongruent Stop trials as well as for left and right side stimuli.
